# Academic Self-Efficacy Partially Mediates the Relationship between Scottish Index of Multiple Deprivation and Composite Attainment Score

**DOI:** 10.3389/fpsyg.2017.01899

**Published:** 2017-11-07

**Authors:** John L. Perry, Martin Dempster, Michael T. McKay

**Affiliations:** ^1^Department of Psychology, Mary Immaculate College, Limerick, Ireland; ^2^Department of Psychology, Queen's University Belfast, Belfast, Ireland; ^3^Department of Psychological Sciences, University of Liverpool, Liverpool, United Kingdom

**Keywords:** Scotland, attainment, Tariff Score, academic self-efficacy, heavy episodic drinking, school attendance

## Abstract

A developing literature continues to testify to the relationship between higher socio-economic status (SES) and better academic attainment. However, the literature is complex in terms of the variety of SES and attainment indicators used. Against the backdrop of a Scottish Government initiative to close the attainment gap between higher and lower SES children, the present study examined the relationship between individual-level Scottish Index of Multiple Deprivation (SIMD) and National Lower Tariff Score in school children in the West of Scotland. Results showed a practically significant relationship between SIMD and Tariff Score. This relationship was partially mediated by higher academic self-efficacy, so that higher belief in academic competency partially mediated the SIMD-Tariff Score relationship. Further, this partial mediation was robust to the influence of gender, sensation seeking, level of school attendance and past month frequency of Heavy Episodic Drinking. It is suggested that increasing attendance and perceived academic competence are viable ways (among others) of attempting to close the attainment gap.

## Introduction

Previous studies have evidenced inequalities in educational outcomes within Scotland. A 2014 study (Sosu and Ellis, 2014) found that by age 12–14 more than twice as many pupils from the most affluent areas performed well in numeracy compared to pupils from the most deprived areas. Relatedly, the Scottish Survey of Literacy and Numeracy (Scottish Government, 2013) observed an attainment gap of 14–17% for reading, 21% for writing, and 12–28% for numeracy in children in primary and secondary school.

According to Becker and Tuppat (2013), “social inequality” refers to “systematic differences in chances for certain favorable outcomes between social categories” (p. 738). Based on this definition it is clear that risks for underachievement across a range of domains, including academic, are not randomly distributed, but are more strongly felt in certain social categories. A substantial educational research literature suggests that social class is the most important variable influencing educational achievement, and that other factors influencing achievement may vary depending on children's social backgrounds (see for example Cassen and Kingdon, 2007; Raffo et al., 2007; Kerr and West, 2010; Lloyd, 2011). Empirical results show that children from low socio-economic status (SES) backgrounds are less likely of progressing to further and higher education than children from high SES families, even when their academic performance is taken into account (e.g., Jackson et al., 2007; Kloosterman et al., 2009).

While there is broad agreement that there is a negative relationship between lower SES and academic attainment (e.g., Kirsch et al., 2002; Mullis et al., 2004; Sirin, 2005; Micklewright and Schnepf, 2007; van Ewijk and Sleegers, 2010), this relationship is complex. It is complicated on three levels; the nature of the measure of SES used, the nature of the measure of attainment used, and the inclusion (or not) of moderating or mediating factors in assessment models (White, 1982; Sirin, 2005). Indeed, there is little or no consensus on the conceptual meaning of SES, or on the optimal way to measure it (Bornstein and Bradley, 2003). Depending on the choice of measures used to assess them, the relationship between SES and attainment has variously been reported as strong (e.g., Sutton and Soderstrom, 1999), or moderate to non-significant (e.g., Ripple and Luthar, 2000).

In a review of the literature, Sirin (2005) highlighted four potential means of assessing or determining SES; parental education, parental income, parental occupation and home resources. The latter of these assesses issues such as an individual's access to computers, books, and the number of bedrooms in the family home. Accordingly, SES based on a home resources approach typically uses a composite score, based on a number of questions, in order to determine the availability of financial resources in the family more broadly. Entitlement to free school meals (FSM) is another proxy measure for SES that has been widely used. Entitlement to FSM has been described as an imperfect proxy for low-income families, and therefore SES (Hobbs and Vignoles, 2007), and is said to be conceptually problematic. The process of determining eligibility is vulnerable to mistakes, and the impact that participation in a school lunch program itself could have on students' school attainment is difficult to differentiate from the impact of SES (Sirin, 2005).

Studies elsewhere have used aggregation techniques to assess SES, based on the particular school that the student attends (e.g., Caldas and Bankston, 1997), or the particular neighborhood where the student lives (Brooks-Gunn et al., 1997). At a school level, it is not uncommon for an aggregated approach to use the overall proportion of pupils at a given school who are entitled to FSM as a broad indicator of the SES for all pupils at that school. At a local level, indices of multiple deprivation (IMD) are widely used. Attributing a “low” SES to all pupils at a school wherein 50% of pupils are entitled to FSM may be “relatively” meaningful (for example if < 5% of pupils in all other schools in the study are entitled to FSM), but even in that example there is a 50–50 chance of it being so attributed erroneously. Additionally, the location of the school is also said to be important, both in terms of the IMD location, and its location in a rural, suburban or urban setting (Sirin, 2005). Use of aggregated measures can lead to the ecological fallacy—the assumption that all students from a given school population (school) share the “general” characteristics of that school. As well as individual (or family) level SES impacting educational attainment, there is evidence that the broader socio-demographic context also contributes to the relationship, and that an interaction between individual-level and broader socio-demographic factors adversely impacts on attainment. Indeed, studies have shown that schools whose population is made up of a high proportion of low-SES pupils perform less well than would be expected based on the quality of the school, with the reverse true for schools with a relatively high average-SES pupil intake (van Ewijk and Sleegers, 2010).

For Pokropek et al. (2015), two issues remain unclear based on the totality of the literature. Firstly, conceptual issues about the nature and meaning of various SES indicators. Secondly, issues about how SES can be reliably measured across countries. Indeed, one of the problems identified in this literature is the fact that terms are used interchangeably to describe social class, status, deprivation, poverty, or a family's ranking on the social ladder (e.g., Sirin, 2005; Rindermann and Baumeister, 2015). For these reasons, studies have reported widely varying effect sizes for the relationship between SES and academic outcomes. In a review of the literature, Sirin (2005) examined the average effect sizes for the relationship between SES and attainment, and reported that the use of parental occupation, income and education yielded broadly similar effect sizes (0.28 ≤ *d* ≤ 0.30), with entitlement to FSM slightly higher (*d* = 0.33), but home resources considerably higher (*d* = 0.51). Similarly, there were differences in effect sizes based on the academic measure used, with the lowest effect size observed for general or composite achievement outcomes (*d* = 0.22), and larger effect sizes for subject-specific outcomes (0.27 ≤ *d* ≤ 0.35). However, it should be pointed out that all of these effect sizes fall short of Ferguson's (2009) threshold for “practical” significance (*d* ≥ 0.41). Indeed, Sirin (2005) more broadly discovered that the “medium” level of association between SES and academic achievement at an individual level, fell short of the “large” degree of association at the school level. While some evidence has suggested that educational inequalities have been in decline, (e.g., Breen et al., 2009), others have evidenced their continued existence (e.g., Pfeffer, 2008). The present study chose Scottish Index of Multiple Deprivation (SIMD) as the measure of SES as it is a Nationally recognized measure, and is not open to participant reporting bias.

Self-efficacy is defined as “people's judgments of their capabilities to organize and execute courses of action required to attain designated types of performance” (Bandura, 1986, p. 391). These beliefs help determine individuals' choices, efforts, persistence, and perseverance in tasks. Self-efficacy is a domain specific construct (e.g., Grau et al., 2001; Muris, 2001; Habel, 2009) such that self-efficaciousness in one domain (e.g., sport) does not always translate to all domains of life (e.g., emotions); therefore, feelings of competence tied to task demands of a given situation have greater predictive utility than a global self-evaluation (Bandura, 1997). According to Graham and Weiner (1996), perceived self-efficacy consistently predicts behavioral outcomes more than any other motivational construct. In their meta-analysis, Multon et al. (1991) found that self-efficacy beliefs accounted for 14% of variance in students' academic performance and 12% of variance in their academic persistence. Self-efficacy is also “associated with key motivational constructs like causal attributions, self-concept, optimism, achievement goal orientation, academic help-seeking, anxiety, and value” (Usher and Pajares, 2008, p.751) and is potentially the most important construct in social cognitive theory.

For many children and young people who have had experiences of adversity or disadvantage, resilience is considered a particularly important factor in academic success (OECD, 2011), and resilient students have been found to have higher levels of self-efficacy (Shumow et al., 1999; Borman and Overman, 2004). In a report funded by the Joseph Rowntree Foundation, a British social policy research and development charity, Hirsch (2007) summarized the findings of eight research studies into the experiences of children from different SES backgrounds and their attitudes to education, and claimed that children from disadvantaged backgrounds were more likely to perceive a lack of control over their learning and were more likely to feel anxious and unconfident about school. The report suggests that these factors are “at the heart of the social divide in educational outcomes” (p. 1). Horgan's study (Horgan, 2007) into the impact of poverty on children's experiences of school found evidence of boys in particular starting to disengage from school from the age of 9 or 10; like Horgan (2007), Archer and Francis (2007) have also pointed out the intersecting nature of gender, and social class (and race) in terms of explaining variance in achievement (also see Heath and Brinbaum, 2007; Breen et al., 2010).

Boggiano et al. (1988) investigated perceptions of competence and ability in children. They found that higher perceptions of competence or abilities resulted in greater preference to engage in challenging learning activities. In addition to its relationship with motivation, research suggests a significant influence of self-efficacy on academic attainment (Zimmerman et al., 1992) and meaningful cognitive engagement (Walker et al., 2006). Indeed, Caprara et al. (2011) recently found that both academic self-efficacy and personality traits individually contributed significantly to academic achievement in school. Furthermore, in a series of large scale studies with secondary school pupils, Trautwein et al. (2009) illustrated that competency beliefs (similar to self-efficacy) and conscientiousness were independent predictors of secondary school pupils' academic effort and achievement. However, there is generally more research examining the relationship between self-efficacy and attainment (e.g., Trautwein et al., 2009; Caprara et al., 2011) than motivation (Boggiano et al., 1988). In a recent study the authors reported that overall, intrinsic and extrinsic academic motivation were best predicted by self-efficacy (McGeown et al., 2014), compared to scores on the Big 5.

The present study matched data provided from a Local Authority in the West of Scotland with data gathered as part of a longitudinal study into alcohol-related behaviors to examine the relationship between SIMD and composite academic attainment, and the degree to which gender, school attendance, sensation seeking, Heavy Episodic Drinking (HED) and scores on three domains of self-efficacy mediate or moderate that relationship. Although our primary research focus was the relationship between SIMD, academic self-efficacy and attainment, we included a number of other variables assessed in the longitudinal study in the present analyses. Sensation seeking is broadly understood as the desire for intense and novel experiences (Zuckerman, 1994), and has been shown to be inversely related to academic performance (Aluja-Fabregat and Torrubia, 1998; Eklund and Fritzell, 2013; Cladellas et al., 2017), in some cases, particularly in boys (Aluja-Fabregat and Torrubia, 1998). Moreover, the Scottish Government have reported that a greater proportion of females than males remain in school beyond compulsory education, and that, on average, females have better academic attainment than males. Finally, with data having been gathered on Heavy Episodic Drinking (HED), this variable was also added to the measurement model. Research across the world has attested to the deleterious effect of alcohol use on academic outcomes in adolescence (e.g., Latvala et al., 2014; Nelson et al., 2015; Patte et al., 2017).

To examine the mediating and moderating roles of self-efficacy, sensation-seeking, and demographic variables, we posit three specific research questions (RQs):

1. Is Tariff Score predicted by SES?
2. To what extent is this relationship mediated by academic, social, and emotional self-efficacy?
3. What is the impact of sensation seeking, gender, attendance and Heavy Episodic Drinking (HED) on observed relationships?

## Methods

### Participants

Participants (*n* = 546) were pupils from five High schools in a Local Authority in the West of Scotland (Male, *N* = 240, 45.8%). Schools committed to participation in the study independently (not mandated by the Local Authority). Ethical approval was obtained from a UK higher education institution and written, informed parental consent was provided for each participant. Data were collected at four time points; baseline (when the students were in the first year of High school; mean age = 12.5 years); and at +12 months; +24 months; and +33 months (when the students were in their fourth year, and just prior to the National Lower examinations).

### Measures

The Self-Efficacy Questionnaire for Children (SEQ-C; Muris, 2001) assesses three domains of self-efficacy: (a) academic self-efficacy (e.g., “How well do you succeed in passing all subjects?”), (b) emotional self-efficacy (e.g., “How well can you control your feelings?”), and (c) social self-efficacy (e.g., “How well do you succeed in staying friends with other children?”). Each subscale contains seven items, to which respondents are required rate their competence in each self-efficacy domain on a 5-point Likert scale anchored by 1 (*not at all*) and 5 (*very well*). The structural validity and internal consistency of SEQ-C subscale scores has previously received robust support (α > 0.80; Muris, 2001). Scores on each domain range from a low of seven to a high of 35.

The Brief Sensation Seeking Scale-4 (BSSS-4; Stephenson et al., 2003) was used to assess self-reported sensation seeking. Respondents were asked how strongly they agreed with four statements describing sensation seeking (e.g., “I would like to explore strange places”). A five-point Likert scale (1 = *strongly disagree* and 5 = *strongly agree*) was averaged within subjects, creating a mean sensation seeking score. The scale has reasonable internal consistency (α ≈ 0.66) (Stephenson et al., 2003; Vallone et al., 2007; α_currentstudy_ = 0.78).

For Heavy Episodic Drinking, participants were asked about the frequency of consuming 5 or more full drinks in the same drinking session in the past month, which was dichotomized for the purpose of this study. Missing data on HED drinking at baseline (T0) were replaced by a whole sample mean imputation. Such imputation has been shown to produce unbiased parameter estimates under certain conditions (see White and Thompson, 2005).

Information was also gathered on gender. Data were then matched with attainment data provided by the Local Authority which included data on school attendance, SIMD for each child, as well as Tariff Score in the Statutory, National Lower Examinations. The Tariff Score of a pupil is a composite attainment score calculated by adding together the weighted Tariff points which he/she accumulates from all the different course levels and awards he/she attains. See Appendix in Supplementary Material for the Tariff Score formula.

Tariff Score refers to the composite score, based on assessment across a broad range of academic subjects. SIMD is calculated by the Scottish Government (Scottish Government, 2016a). Using a variety of indicators (e.g., level of crime, travel time to the nearest General practitioner, level of unemployment), Scotland is partitioned into 6,976 data zones which are ranked, and clustered into deciles. A SIMD of 10 indicates living in the top decile, with a SIMD of 1 indicative of living in the bottom decile.

### Data analyses

Preliminary analyses screened for missing data, outliers, and normality before assessing the internal consistency of each scale. Internal consistency was estimated by omega point estimates and confidence intervals, as omega holds fewer assumptions about the scale and sampling than alpha (Dunn et al., 2013). We then examined the factor structure of each scale to ensure it was appropriate in the study sample.

For the main analyses, we examined each Research Question (RQ) in turn through path analysis. RQ1 required a simple path analysis to determine the extent to which Tariff Score is predicted by SES. Thereafter, each RQ was examined by adding mediating and/or moderating variables to the path model preceding it.

## Results

### RQ1: is tariff score predicted by SES?

Firstly, we examined if SIMD was stable over time. To do so, a repeated measures ANOVA was used to test changes at each of the four time periods (T1 = 4.10, T2 = 4.08, T3 = 4.11, T4 = 4.11). Correcting for sphericity [Mauchly's *W*_(5)_ = 0.196, *p* ≤ 0.001], ANOVA revealed no significant change across time [*F*_(1.63)_ = 0.271, *p* = 0.72]. Consequently, it was appropriate to calculate and use mean SIMD in our subsequent analyses. We next examined the descriptive statistics and distribution for mean SIMD and Tariff Score (Table 1). The descriptive analyses indicated no concerns with outliers or normality (skewness and kurtosis ≤ 2).

**Table 1:** Descriptive statistics for mean Scottish Index of Multiple Deprivation and Tariff Score.

**Variable**	**Mean**	**SD**	**Median**	**Min**	**Max**	**Skew**	**Kurt**	**Variance**
SIMD (*n* = 546)	4.13	2.70	3.00	0.00	10.00	0.44	−1.16	7.31
Tariff Score (*n* = 521)	428.84	165.51	424.00	18.00	756.00	−0.15	−0.68	28060.21

**Figure 1:**
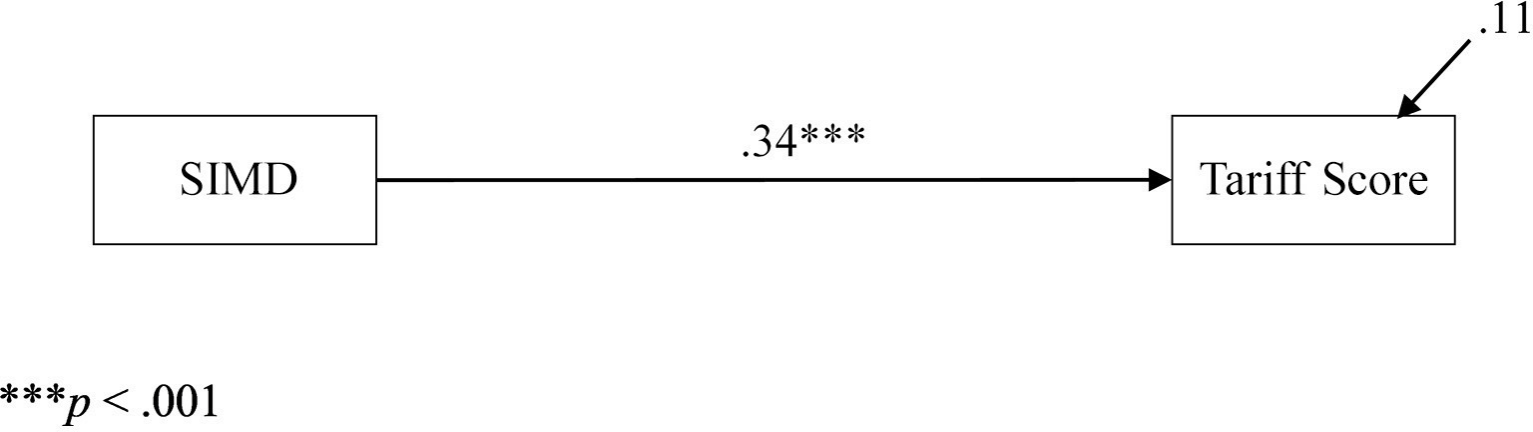
Path model from SIMD to grade with standardized estimates.

All path analyses were conducted in Mplus 7 (Muthén and Muthén, 1998-2012) with the maximum likelihood estimator and 5,000 bootstrapped samples to generate 95% confidence intervals. The first, simplest model examined was to determine if Tariff Score was statistically predicted by mean SIMD (Figure 1). Indeed, the single path in this model was statistically significant (β = 20.93, *p* ≤ 0.001, 95% CI = 13.97, 27.67, β = 0.34).

### RQ2: to what extent is this relationship mediated by academic, social, and emotional self-efficacy?

The next model sought to examine the hypothesized mediating effect of academic, social, and emotional self-efficacy on Tariff Score. Before calculating path estimates however, it is good practice to examine the validity and reliability of the scale used to measure self-efficacy. The 21-item, three-factor scale used identified seven items for each of the three factors; academic self-efficacy (ASE), social self-efficacy (SSE), and emotional self-efficacy (ESE). To test the factor structure of the scale, we conducted exploratory structural equation modeling (ESEM; Asparouhov and Muthén, 2009) on the scale at each of the four time points. In ESEM models, all observed variables are loaded onto a pre-determined number of latent variables. This is advantageous compared to traditional confirmatory factor analyses of independent cluster models (CFA-ICM) because non-significant or negligible cross-loadings are not viewed as mis-specifications (Marsh et al., 2004). All ESEM analyses employed the maximum likelihood estimator. We assessed model fit using incremental indices; comparative fit index (CFI) and Tucker-Lewis index (TLI) and absolute indices; root-mean-square error of approximation (RMSEA) and standardized root mean-square residual (SRMR). Model fit was cautiously interpreted as adequate if CFI and TLI were close to 0.90, and RMSEA and SRMR were close to 0.05 and 0.08 respectively, as suggested by Hu and Bentler (1999). We recognize however, the recommendations of Perry et al. (2015) to avoid rigidly using these as cut-off values. At each time point, the self-efficacy scale presented acceptable factorial validity (Table 2).

**Table 2:** Model fit indices for self-efficacy scale using Exploratory Structural Equation Modeling.

**Time**	**χ^2^**	***df***	**CFI**	**TLI**	**SRMR**	**RMSEA (90% CI)**
T1	327.15	150	0.930	0.901	0.036	0.051 (0.043, 0.059)
T2	392.41	150	0.929	0.900	0.033	0.058 (0.051, 0.065)
T3	517.59	150	0.911	0.875	0.036	0.072 (0.066, 0.079)
T4	506.09	150	0.915	0.881	0.038	0.072 (0.066, 0.079)

All self-efficacy scales presented no issues with outliers or normality (skewness ≤ 1, kurtosis ≤ 1). We next examined the internal consistency of ASE, SSE, and ESE using omega point estimates and confidence intervals. Omega point estimates and confidence intervals were calculated using the MBESS package (Kelley and Lai, 2012), in R (R Development Core Team, 2012), with 1,000 bootstrapped samples. This was conducted for the three self-efficacy subscales at each of the four data collection points. All scales demonstrated acceptable internal consistency throughout all time periods (Table 3).

**Table 3:** Omega point estimates and confidence intervals for self-efficacy scores at all-time points.

**Scale**	**T1**	**T2**	**T3**	**T4**
Academic	0.85 (0.83, 0.87)	0.78 (0.74, 0.81)	0.88 (0.86, 0.90)	0.87 (0.85, 0.90)
Social	0.69 (0.63, 0.73)	0.71 (0.66, 0.75)	0.79 (0.75, 0.82)	0.79 (0.75, 0.83)
Emotional	0.78 (0.75, 0.81)	0.71 (0.66, 0.76)	0.79 (0.75, 0.82)	0.90 (0.88, 0.91)

Having established the validity and internal consistency of the self-efficacy scale, we next examined the extent to which this changed over time, conducting a repeated-measures ANOVA. Descriptive statistics appeared to demonstrate very little meaningful change over time (Table 4). Tests of within-subject effects were corrected for sphericity [ASE Mauchly's *W*_(5)_ = 0.837, *p* ≤ 0.001; SSE *W*_(5)_ = 0.823, *p* ≤ 0.001; ESE *W*_(5)_ = 0.841, *p* ≤ 0.001]. Although univariate tests revealed statistically significant effects for the decrease in ASE [*F*_(2.66)_ = 35.14, *p* ≤ 0.001] and SSE [*F*_(2.64)_ = 5.09, *p* ≤ 0.01], these were practically negligible. Using Ferguson's (2009) recommendations for minimum practical effect, only the difference between ASE from T1 to T4 identifies a meaningful effect size (*d* = 0.44). All other effect sizes presented Cohen's *d* ≤ 0.41. Overall, there is little change in self-efficacy across time points. Given that it is the closest to the Tariff Score calculation, the self-efficacy scores from T4 were used in the proceeding path analyses.

**Table 4:** Mean and standard deviation statistics for self-efficacy at all-time points.

**Scale**	**T1**	**T2**	**T3**	**T4**
Academic	25.43 (5.07)	24.48 (5.58)	23.91 (5.30)	23.16 (5.57)
Social	25.82 (4.24)	25.72 (4.28)	25.06 (4.74)	25.39 (4.68)
Emotional	21.73 (5.08)	21.53 (5.76)	21.09 (6.05)	21.40 (6.08)

**Figure 2:**
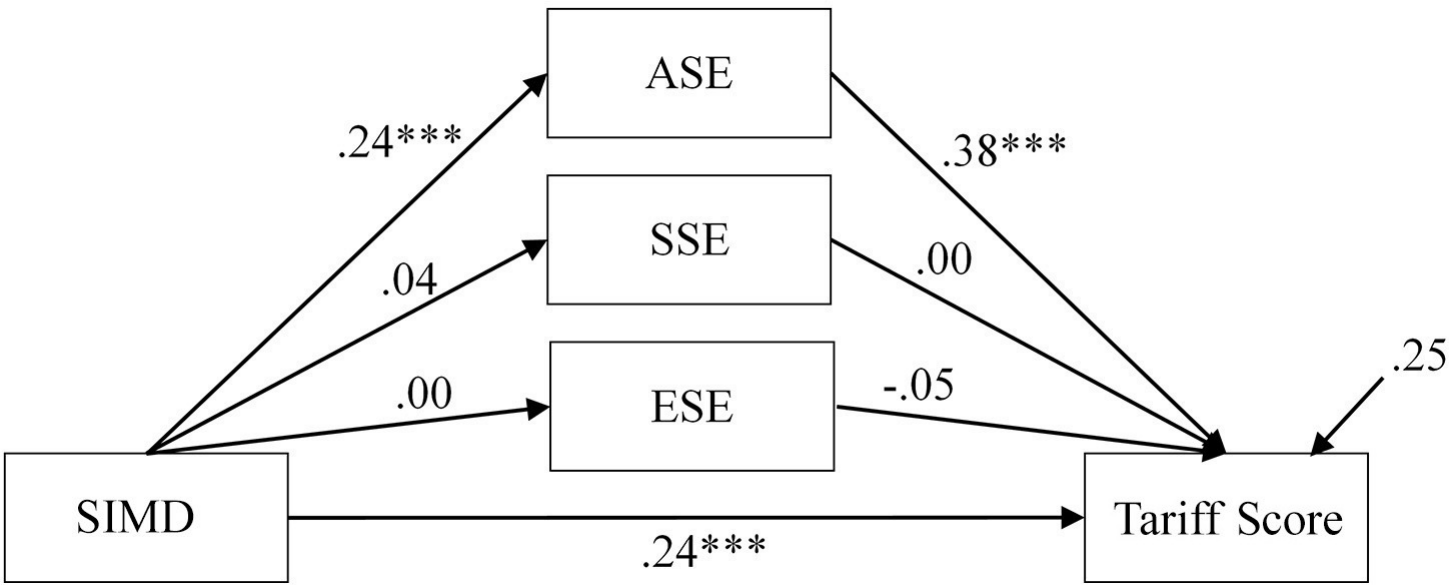
Combined effects path model showing standardized estimates. ^***^p < 0.001.

We next examined the extent to which the established positive relationship from RQ1 between SIMD and Tariff Score was mediated by self-efficacy. This comprised of an iterative process to firstly test a combined effects model that included all three efficacy scales and the direct path from SIMD to grade (Figure 2).

**Figure 3:**
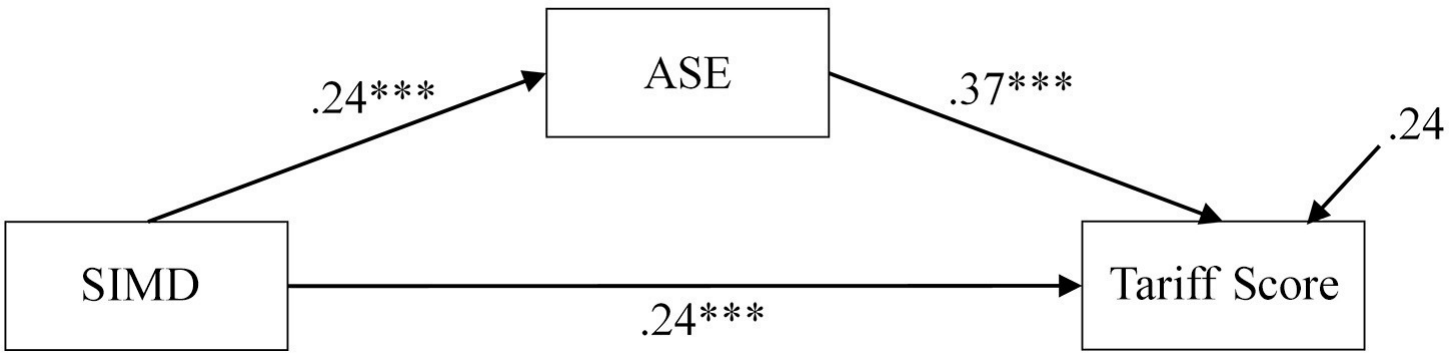
Path model for simplified combined effects model showing standardized estimates. ^***^p < 0.001.

The combined effects model contained a significant path from SIMD to Tariff Score, which indicates that partial mediation is present (β = 15.01, *p* ≤ 0.001, 95% CI = 8.41, 21.81, β = 0.24). The path from SIMD to ASE was positive and significant (β = 0.51, *p* ≤ 0.001, 95% CI = 0.25, 0.76, β = 0.24), as was the path from ASE to Tariff Score (β = 11.26, *p* ≤ 0.001, 95% CI = 7.37, 14.83, β = 0.38). No other significant direct effects were present. The indirect effect from SIMD to Tariff Score via ASE was significant (γ = 5.71, *p* ≤ 0.001, 95% CI = 2.67, 9.24, γ = 0.09). It was clear that SSE and ESE had no effect on the relationship between SIMD and Tariff Score. As such, these were removed for parsimony and the model re-examined (Figure 3).

### RQ3: what is the impact of sensation seeking, gender, attendance and HED on observed relationships?

As before, we examined sensation seeking (SS) for outliers and normality, identifying no issues (skewness ≤ 1, kurtosis ≤ 1). A repeated-measures ANOVA, corrected for sphericity [Mauchly's *W*_(5)_ = 0.966, *p* ≤ 0.01], suggested a significant change in SS over time [*F*_(2.93)_ = 13.50, *p* ≤ 0.001]. These effects however were negligible, as mean varied very little across time (T1 = 14.96, T2 = 14.79, T3 = 14.37, T4 = 14.22). Indeed, even the largest pairwise comparison (T1 to T4) represented an effect size of only *d* = 0.22. Overall, there is no evidence of a change in the propensity for SS over the four time periods. As such, the mean of the four measurements was used in the subsequent path analyses.

**Figure 4:**
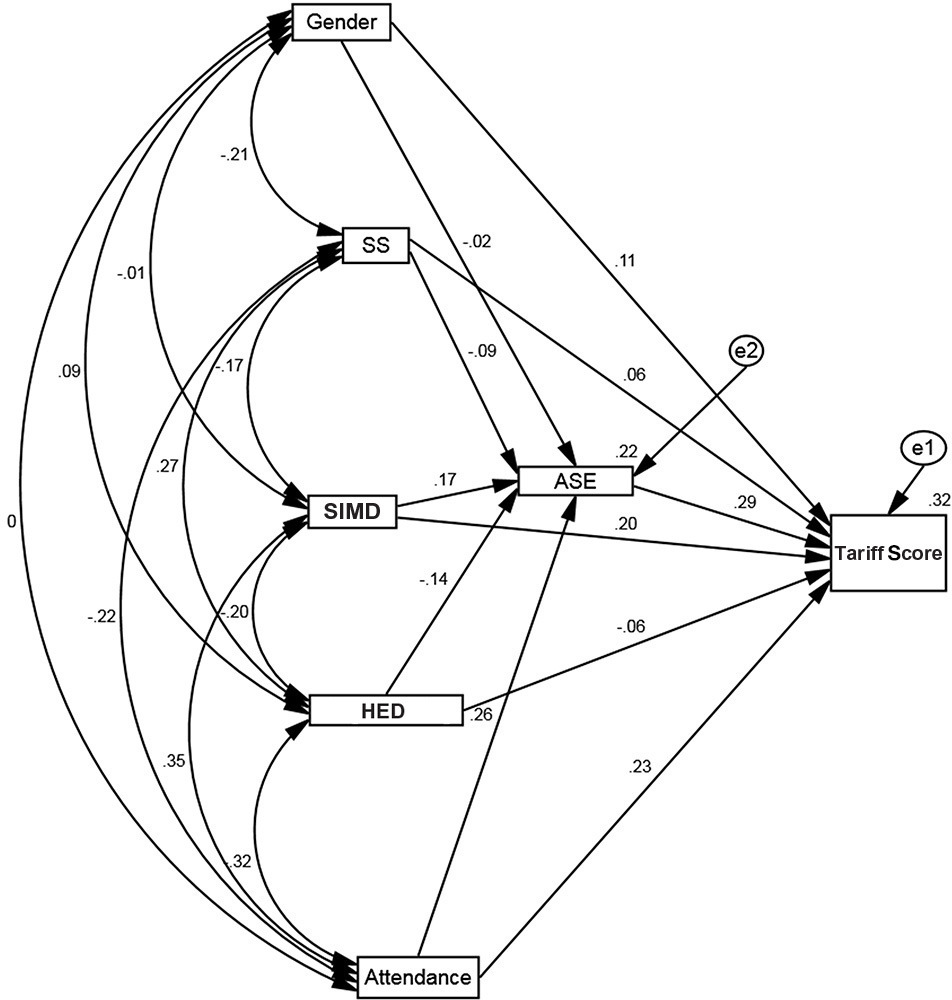
Path analysis including SS, attendance, HED, and gender as additional effects.

In an exploratory analysis, SS, school attendance, gender and Heavy Episodic Drinking (HED) were all added to the model and the direct and indirect (via ASE) effects examined, along with the potential moderating effect of these variables on the relationship between SIMD and Tariff Score. The standardized path coefficients for these moderating effects were very small, ranging from 0.04 to 0.08, so these were removed from the model (Figure 4).

**Figure 5:**
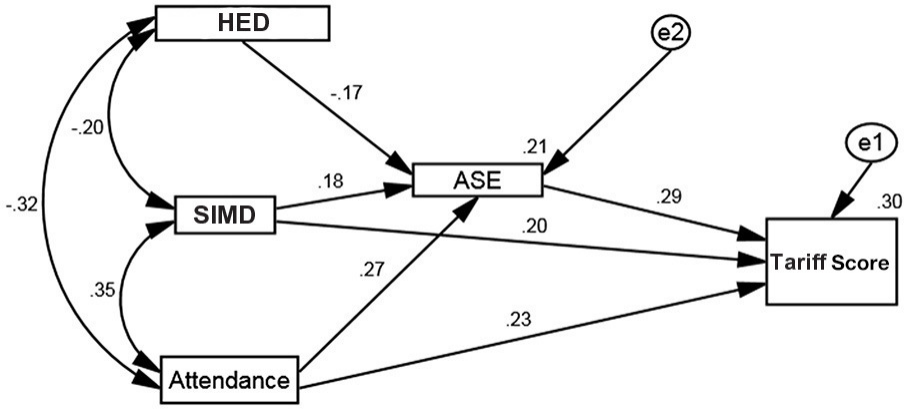
Final, most parsimonious, path model.

In an attempt to find the most parsimonious model, the path with the smallest standardized coefficient was removed from the model in Figure 4 and the path coefficients recalculated. This process was continued in an iterative way until the most parsimonious model was defined (i.e., the model with the smallest number of parameters, without a substantial decrease in the variance explained by the model). The final model is specified in Figure 5.

## Discussion

The present study sought to investigate the degree to which SES (assessed by SIMD) predicts Tariff Score (composite academic score) in a sample of Scottish school children, the degree to which that relationship is mediated by (academic) self-efficacy, and whether or not those relationships were robust to the influence of sensation seeking, school attendance, HED and gender. While the simple answer is that academic self-efficacy does partially mediate the relationship between SES and attainment (robust to the influence of the above-mentioned variables), the picture that emerged was both instructive and complex.

Using Ferguson's (2009) criteria for a recommended minimum interpretable effect size (practically significant, β ≥ 0.20) results show practically significant positive associations between Tariff Score and academic self-efficacy, higher SES, and a higher level of school attendance. While SIMD was significantly associated with Tariff Score in the final model, the coefficients for attendance and academic self-efficacy were larger. Essentially this suggests that while there may be some patterning of Tariff Score by SIMD, this can be overcome if individuals attend school more frequently and have a higher level of belief in their own academic competency. This is an interesting and potentially useful finding as these variables (higher attendance and a greater self-belief in academic ability) can be enhanced through mentoring and within-school support structures. However, it is possible that variables not measured in this study, including parental educational attainment, parental employment status and parental views on education, could be additional mediators in this model. For example, it is highly likely that school attendance is positively influenced both by parental rule setting and parents' own experiences of, or belief in, education. These variables were not measured in the present study, and this is a limitation.

While Figure 4 shows that sensation seeking, gender, and HED did not have a practically significant effect on Tariff Score, they did have an indirect influence. So, while it may be tempting to conclude that HED has no influence at all on Tariff Score this is, probably not true. For example, looking at school attendance, it is clear that sensation seeking and HED are both negatively associated with school attendance, with SES positively associated with attendance. Said another way, while the results seem to suggest that Tariff Score is not directly influenced by HED, it is not legitimate to conclude that adolescent drinking has absolutely no impact on attainment, with the present results suggesting that the effect may be seen via school attendance.

The present study should be understood in the context of some limitations. Firstly, the Tariff Score is a composite score based on scores across a series of subjects, and there is some individual-level variation on these subject choices. It is possible that global achievement measures conceal differences among subject areas (for example, arithmetic and written achievement) and therefore may obscure meaningful differences between subjects. Secondly, while the attendance, SIMD, and Tariff Scores were supplied by the Local Authority, HED, sensation seeking, and self-efficacy scores were self-reported. Finally, only five schools participated, and results may not generalize to other locations. Further research will be required to determine the extent to which they do.

Recently, the Scottish Government outlined four key priorities in its National Improvement Framework (Scottish Government, 2016b), one of which was “closing the attainment gap between the most and least disadvantaged children” (p. 2). In a context where SIMD continues to be positively related to educational attainment, the present study suggests that improvement in two individual-level areas could potentially impact on closing this gap, namely encouraging children to attend school more frequently, and enhancing their belief in their ability to achieve academically. These are not simple options, but are more viable in the immediate future than changing SIMD. However, it is likely that the reasons for non-attendance, and a lack of belief, are also influenced by factors such as previous negative reinforcement, and apathy at an individual and family level with regard to education. The present results suggest that if deliberate efforts are made to encourage attendance and to raise efficacy, there are potential attainment benefits for those concerned. Elsewhere, five important approaches have been suggested which could assist in boosting individuals' academic self-efficacy, namely: helping students to set clear and specific goals, encouraging the use of challenging and proximal goals, provision of honest feedback to increase efficacy beliefs, assisting students with the accurate calibration of self-efficacy, and the use of peer modeling to build self-efficacy (Artino, 2012).

## Ethics statement

Ethical approval was granted by The Centre for Public Health, Liverpool John Moores University, providing consent for the use of human participants for this study.

## Author contributions

MM organized the study and data collection. JP performed statistical analysis and aided the conceptualization of study. MD performed further statistical analyses. All authors contributed to writing the manuscript.

### Conflict of interest statement

The authors declare that the research was conducted in the absence of any commercial or financial relationships that could be construed as a potential conflict of interest.
