# Supplementary material for: Academic Self-Efficacy Partially Mediates the Relationship between Scottish Index of Multiple Deprivation and Composite Attainment Score
Source: Front Psychol. 2017 Nov 7;8:1899. doi: 10.3389/fpsyg.2017.01899 (PMC5681956; doi:10.3389/fpsyg.2017.01899)
Supplement: Supplementary file 1 [file Appendix.pdf]

Appendix A

The tariff points for a unit or course are currently calculated as follows:

Tariff points = (CP * multiplier) + ((CP * (course wt + result wt)) / NCC)

Where:

- CP = Scottish Credit and Qualifications Framework (SQCF) credit points
- Multiplier. This is the multiplier for the SCQF level.
- Course wt = Course weight. This is the weight for attaining the full course at that SCQF level (0 for a single unit or combination of units making up an ungraded course, or a variable weight if a course assessment element is involved i.e. added value unit at National 4 or course assessment at National 5 and above).
- Result wt = Result weight. This is the weight which differentiates between the grade achieved (A, B, C, D) for the course at the SCQF level. The weight is such that a grade D receives more points than units alone, recognising that a learner has achieved the course.
- NCC = National course correction. This is the SCQF credit points for the SQA National Course at the same level (with the exception of the BAC IP)

Available at: http://insight-guides.scotxed.net/support/InsightTariff.pdf
